# The CXCR1/CXCR2 Inhibitor Reparixin Alters the Development of Myelofibrosis in the *Gata1* ^low^ Mice

**DOI:** 10.3389/fonc.2022.853484

**Published:** 2022-03-22

**Authors:** Paola Verachi, Francesca Gobbo, Fabrizio Martelli, Andrea Martinelli, Giuseppe Sarli, Andrew Dunbar, Ross L. Levine, Ronald Hoffman, Maria Teresa Massucci, Laura Brandolini, Cristina Giorgio, Marcello Allegretti, Anna Rita Migliaccio

**Affiliations:** ^1^ Department of Biomedical and Neuromotor Sciences, Alma Mater Studiorum University, Bologna, Italy; ^2^ Department of Veterinary Medical Sciences, University of Bologna, Bologna, Italy; ^3^ National Center for Drug Research and Evaluation, Istituto Superiore di Sanità, Rome, Italy; ^4^ Center for Animal Experimentation and Well-Being, Istituto Superiore di Santà, Rome, Italy; ^5^ Human Oncology & Pathogenesis Program, Memorial Sloan Kettering Cancer Center, New York, NY, United States; ^6^ Leukemia Service, Department of Medicine and Center for Hematologic Malignancies, Memorial Sloan Kettering Cancer Center, New York, NY, United States; ^7^ Center for Epigenetics Research, Memorial Sloan Kettering Cancer Center, New York, NY, United States; ^8^ Division of Hematology/Oncology, Tisch Cancer Institute and Department of Medicine, Icahn School of Medicine at Mount Sinai, New York, NY, United States; ^9^ Dompé Farmaceutici Spa R&D, L’Aquila, Italy; ^10^ Center for Integrated Biomedical Research, Campus Bio-medico, Rome, Italy; ^11^ Altius Institute for Biomedical Sciences, Seattle, WA, United States

**Keywords:** myelofibrosis, TGF-β, megakaryocytes, GATA1, CXCL8 (interleukin-8)

## Abstract

A major role for human (h)CXCL8 (interleukin-8) in the pathobiology of myelofibrosis (MF) has been suggested by observations indicating that MF megakaryocytes express increased levels of hCXCL8 and that plasma levels of this cytokine in MF patients are predictive of poor patient outcomes. Here, we demonstrate that, in addition to high levels of TGF-β, the megakaryocytes from the bone marrow of the *Gata1* ^low^ mouse model of myelofibrosis express high levels of murine (m)CXCL1, the murine equivalent of hCXCL8, and its receptors CXCR1 and CXCR2. Treatment with the CXCR1/R2 inhibitor, Reparixin in aged-matched *Gata1* ^low^ mice demonstrated reductions in bone marrow and splenic fibrosis. Of note, the levels of fibrosis detected using two independent methods (Gomori and reticulin staining) were inversely correlated with plasma levels of Reparixin. Immunostaining of marrow sections indicated that the bone marrow from the Reparixin-treated group expressed lower levels of TGF-β1 than those expressed by the bone marrow from vehicle-treated mice while the levels of mCXCL1, and expression of CXCR1 and CXCR2, were similar to that of vehicle-treated mice. Moreover, immunofluorescence analyses performed on bone marrow sections from *Gata1* ^low^ mice indicated that treatment with Reparixin induced expression of GATA1 while reducing expression of collagen III in megakaryocytes. These data suggest that in *Gata1^low^* mice, Reparixin reduces fibrosis by reducing TGF-β1 and collagen III expression while increasing GATA1 in megakaryocytes. Our results provide a preclinical rationale for further evaluation of this drug alone and in combination with current JAK inhibitor therapy for the treatment of patients with myelofibrosis.

## Introduction

Primary Myelofibrosis (MF) is due to both a primary clonal myeloproliferation as a result of activating mutations of the JAK/STAT pathway and a secondary inflammatory response characterized by micro-environmental changes and aberrant release of multiple pro-inflammatory cytokines (1). Abnormal megakaryocytes (MKs) play a crucial role in the development of the MF stromal reaction (2), which includes bone marrow (BM) reticulin fibrosis, osteosclerosis, increased microvessel density, a proinflammatory milieu, anemia, splenomegaly, and extramedullary hematopoiesis (1, 3). The current JAK1/2 inhibitor therapy improves clinical symptoms but does not alter the clinical progression to more overt phases of MF or blast phase (4, 5). Therefore, novel therapeutic strategies aiming to reduce the inflammatory microenvironment that contributes to the sustained proliferation of the malignant hematopoietic stem cells (HSCs) are currently being investigated (6).

Human (h)CXCL8 (C-X-C Motif Chemokine Ligand 8) is a member of the chemokine family and exerts its biological activities by signaling through the CXCR1 and CXCR2 receptors. The chemokine family also includes CXCL1, CXCL2, CXCL3, CXCL5, CXCL6, and CXCL7, and together they share an ELR (glutamic, leucine, and arginine) motif that mediates CXCR1/2 binding. Most of the studies published until now have investigated the biological effects exerted by hCXCL8 and its receptors on polymorphonuclear leukocytes. However, the effects exerted by hCXCL8 on other cell types, such as endothelial, epithelial and fibroblasts, known also to express CXCR1/CXCR2, are still poorly defined (7). hCXCL8 is produced by several bone marrow cells, namely, megakaryocytes (MKs) (8), and exhibits many biological functions in inflammation, hematopoietic stem cell (HSC) proliferation, mobilization, and neo-angiogenesis. A previous study demonstrated that hCXCL8 contributes to altered MK proliferation, differentiation, and ploidy in myeloid metaplasia with MF (9). Moreover, high levels of circulating hCXCL8 were detected in patients with MF and were predictive of inferior survival (3). In addition, Dunbar et al. have demonstrated that the malignant CD34^+^ clones from a subset of MF patients secrete high levels of hCXCL8 *in vitro* which is associated with adverse clinical outcome and increased marrow fibrosis (10).

Full understanding of the pathophysiological role of hCXCL8 has been limited by known differences between human and rodents. In humans, hIL-8/CXCL8 (and CXCL6, also known as GCP-2) exerts its activity by activating both CXCR1 and CXCR2 (11, 12), whereas the other ELR-CXC chemokines selectively bind CXCR2 (13). For many years, only one functional ELR-CXC receptor was identified in mice and was characterized as the homologue of human CXCR2 (14, 15). This receptor, showing high affinity for the murine counterparts of hCXCL8, mCXCL1(KC) and MIP-2, was believed to be responsible for the functions attributed to the two human receptors. Consistent with this, gene ablation of mouse CXCR2 impairs neutrophil responses to murine MIP-2 and to hIL-8/CXCL8 (16, 17) confirming a critical role for CXCR2 in neutrophil recruitment and activation. The orthologous murine CXCR1 (mCXCR1) (18, 19) was subsequently identified and found to be expressed by BM, peripheral mononuclear cells, CD4^+^ and CD8^+^ T cells, and certain lymphoid cell types but was first considered to be a non-functional receptor due to the repeated failure of any attempt to identify its cognate ligand/s. More recently, mCXCR1 has been confirmed to behave as a functional receptor (20), specifically activated by mGCP-2, hGCP-2/CXCL6, and hIL-8/CXCL8, and to play a key role in collagen-induced arthritis (mCIA). This discovery paved the way for novel studies on the biology of CXCR1/2 using mouse models. These studies support the notion that mCXCR1 is the functional murine orthologue of hCXCR1 and that hIL-8/CXCL8 is functionally replaced, in addition to mCXCL1, by CXCL6 in mice (21).

Reparixin is a dual, non-competitive allosteric antagonist of the hCXCL1 receptors hCXCR1/R2 with a marked selectivity for hCXCR1 (IC_50_ = 1 nM for hCXCR1; IC_50_ = 400 nM for hCXCR2) and cross-reactivity with mCXCR1/2 (22, 23). In particular, the allosteric modulation exerted by Reparixin inhibits human and murine CXCR1/R2 activation independently of the cognate ligand (24) and without blocking the binding of the ligand to its receptors (25). The mechanism of action of Reparixin accounts for the functional selectivity of the drug that allows it to switch off the G-protein mediated pathway activation without impairing ligand-induced internalization and scavenging thus not affecting the extracellular levels of mCXCL1. The *in vivo* activity of Reparixin was originally evaluated in animal models of ischemia/reperfusion injury (22) and the therapeutic potential of CXCR1/R2 inhibition was further investigated in the airway inflammation of several mouse models. In particular, Reparixin was shown to ameliorate pulmonary fibrosis caused by the administration of particulate matter and bleomycin in mice (26). Similarly, the CXCR1/R2 inhibition exerted by ladarixin, a dual allosteric blocker of CXCR1/R2 structurally similar to Reparixin, has been shown to reduce neutrophil infiltration and collagen deposition in the bleomycin-induced mouse model of pulmonary fibrosis (27). These data suggest CXCR1/2 inhibition might have anti-fibrotic effects across numerous organs.

Mice carrying the hypomorphic *Gata1* ^low^ mutation express the same MKs alterations observed in MF patients and develop progressive MF closely resembling human disease, namely, a BM failure syndrome and development of extramedullary hematopoiesis (28–30). Previous studies have demonstrated that MKs from the BM of *Gata1* ^low^ mice express not only high levels of TGF-β1 (31), but also high levels of mCXCL1, the murine equivalent of hCXCL8 (32). Using these data as a foundation, in the current study we have evaluated whether MKs from *Gata1* ^low^ mice express CXCR1 and CXCR2 and tested whether treatment with Reparixin affects the development of MF in this mouse model.

## Materials and Methods

### Mice

*Gata1* ^low^ mice were originally obtained from Dr. S. Orkin and bred in the animal facility of the Istituto Superiore di Sanità as described (30). Littermates were genotyped at birth by PCR as previously described (33) and those found not to carry the mutation were used as wild-type (WT) controls. All the experiments were performed according to the protocols approved by the institutional animal care committee according to the European Directive 86/609/EEC.

### Treatment

**Figure 1:**
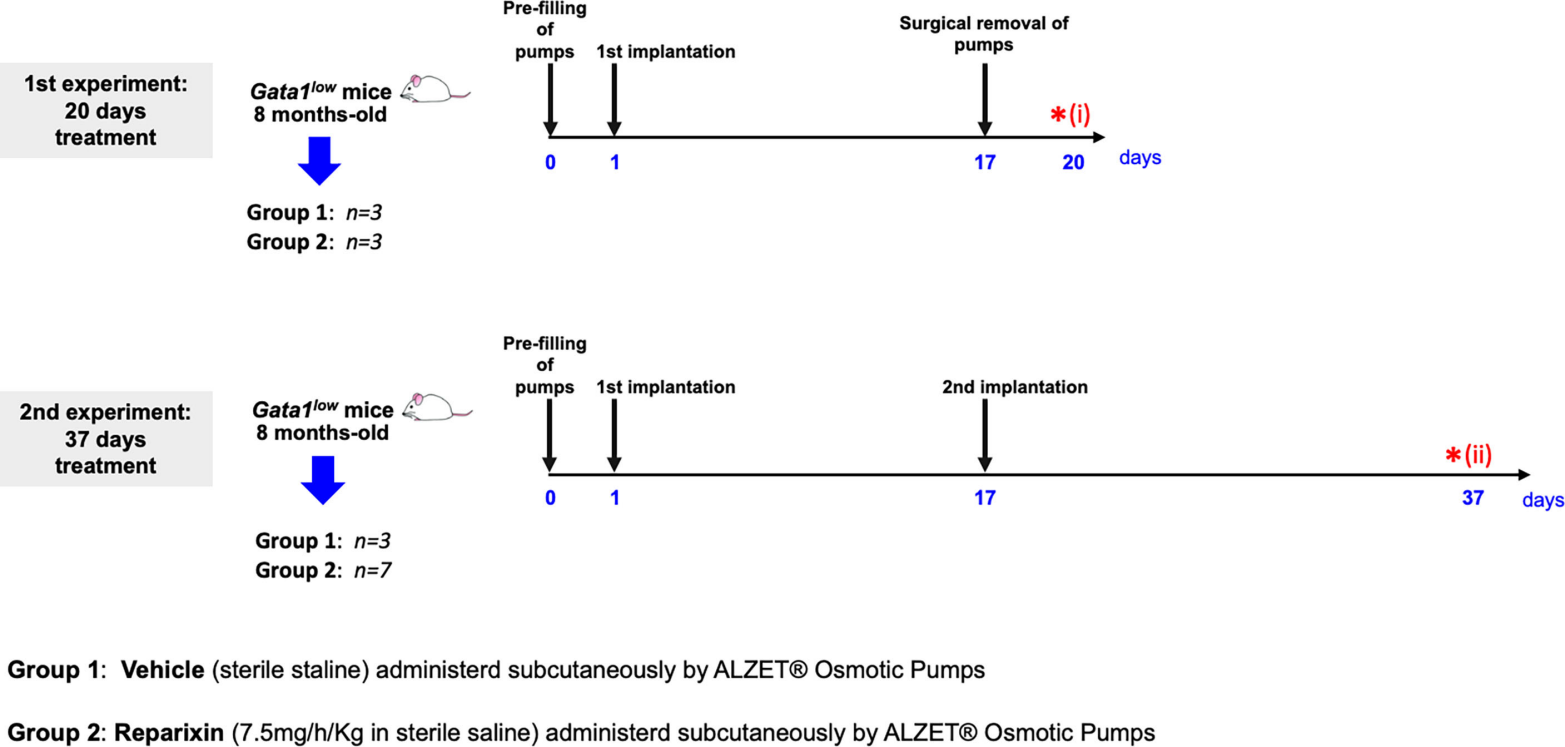
Scheme of the two treatments of *Gata1* ^low^ mice with either vehicle or Reparixin for 20 (experiment 1) or 37 (experiment 2) days. In experiment 1, mini-pumps were removed at day 17 and three mice per experimental group were sacrificed at day 17 for end-point determination. In experiment 2, mini-pumps were removed at day 17 from three vehicles and six Reparixin-treated mice and the mini-pumps were replaced with a second implant. These mice were then treated for 17 additional days and sacrificed at day 37. Red asterisks indicate the timing of the sacrifice in the first (i) and second (ii) experiment.

Sixteen eight-month-old *Gata1* ^low^ mice were then anesthetized with 2–3% isoflurane and implanted subcutaneously with an ALZET^®^ Osmotic Pump (model 2002) pre-filled with 200 μl of vehicle (sterile saline) or Reparixin (7.5 mg/h/kg in sterile saline) as described by the instructions of the manufacturer. The concentration of Reparixin was chosen on the basis of previous concentration–response and efficacy studies in which the selected concentration of 7.5 mg/h/kg administered by continuous infusion was proven to be able to reduce pathological outcomes in preclinical models of liver ischemia and reperfusion, neuropathic pain, and acquired epilepsy (34–36) Before treatment, the mouse genotype had been confirmed by PCR as described (33). The scheme of the two experiments and of their end-points is outlined in **Figure 1**.

In the first experiment, mini-pumps were removed on day 17, and three mice per experimental group were weighed, bled for blood count determinations and plasma collection, and sacrificed for histopathological evaluation of the BM and spleen at day 20. Since the manufacturer guarantees that the Alzet model 2002 mini-pumps deliver the drug with the predicted rates only for 14 days, to mimic the clinical situation in which Reparixin is likely to be administered to patients for long time, in the second experiment, mini-pumps were removed from three mice treated with vehicle and seven mice treated with Reparixin and replaced with newly filled devices. These mice were then treated for 17 additional days and analyzed on day 37, when they were weighed, bled for blood count determinations and plasma collection, and sacrificed for histopathological analyses.

### Blood Collection

Mice were topically anesthetized with lidocaine (one drop/eye) and blood was collected from the retro-orbital plexus into microcapillary tubes using sodium citrate 3.2% (ratio 1:9) as an anticoagulant. Hematocrit (Htc), platelet (Plt), and white blood cell (WBC) counts were evaluated by an accredited commercial laboratory which provides diagnostic services on laboratory animals (Plaisant Laboratory). For drug concentration determinations, plasma was separated from whole blood by centrifugation for 20 min at 10,000 rpm and stored at −20°.

### Determination of the Plasma Concentration of Reparixin

Determinations of Reparixin levels in plasma samples involved protein precipitation by the addition of acetonitrile (Sigma-Aldrich, S. Louis, MO, USA) (ratio 1:3). Samples were then centrifuged (20,000×*g* for 15 min at 4°C) and supernatants were analyzed by high-performance liquid chromatography (HPLC, Dionex-Thermo Fisher Scientific, Sunnivale, CA, USA) using an electrospray ionization (ESI) source for detection. The chromatographic column was a Gemini C18 100 × 2.0 mm, 5 µm (Phenomenex, Torrance, CA, USA) and the lower limit of quantification is 0.05 µg/ml.

### Histological Analyses

Femurs were fixed in formaldehyde (10% v/v with neutral buffer), treated for 1 h with a decalcifying solution (Osteodec; Bio-Optica, Milan, Italy) and paraffin embedded. Spleens were fixed in formaldehyde as previously described (32) and embedded in paraffin. Paraffin-embedded tissues were cut into consecutive 3 μm sections and stained either with Hematoxylin–Eosin (H&E; Hematoxylin cat no. 01HEMH2500; Eosin cat no. 01EOY101000; Histo-Line Laboratories, Pantigliate, MI, Italy) or Gomori silver (cat no. 04-040801; Bio-Optica) or Reticulin (cat no. 04-040802; Bio-Optica) staining. These two last stainings both reveal the presence of reticulin fibers and were used as independent evaluations of fibrosis to increase the rigor of the assessment of the findings. BM sections were immune-stained with anti-CXCL1 (cat# ab86436, Abcam, Cambridge, UK), anti-CXCR1 (cat# GTX100389, Genetex, Irvine, CA, USA), anti-CXCR2 (cat# catalog ab14935, Abcam) or anti-TGF-β1 (cat no. sc-130348, Santa Cruz Biotechnology, Santa Cruz, CA, USA, from now on we use TGF-β1 or TGF-β, depending whether results were obtained with reagents which recognize the TGF-β1 isoform or all the three TGF-β isoforms), antibodies. Immunoreactions were detected with avidin–biotin immune-peroxidase (Vectastain Elite ABC Kit, Vector Laboratories, Burlingame, CA, USA) and the chromogen 3,3′-diaminobenzidine (0.05% w/v). Slides were counterstained with Papanicolaou’s hematoxylin (Histo-Line Laboratories). Images were acquired with an optical microscope (Eclipse E600; Nikon, Shinjuku, Japan) equipped with the Imaging Source “33” Series USB 3.0 Camera (cat no. DFK 33UX264; Bremen, DE). Images were processed and the intensity of the immunostaining quantified with the software ImageJ (version 1.52t) (National Institutes of Health).

### Immunofluorescence Analysis

Three micron-thick BM sections were dewaxed in xylene and treated with EDTA buffer pH = 8 for 20’ in a pressure cooker (110–120°C, high pressure) for antigen retrieval. Sections were labeled with antibody against CD42b (a rat monoclonal antibody that recognizes the alpha chain of platelet glycoprotein I, cat no. ab183345, Abcam), GATA1 (rat monoclonal, cat no. sc-265, Santa Cruz), and Collagen III (rabbit polyclonal, cat no. ab7778, Abcam) overnight at 4°C. Detection of primary antibodies was visualized with a secondary antibody goat anti rat Alexa Fluor 488 (cat no. ab150165, Abcam) and goat anti rabbit Alexa Fluor 555 (cat no. ab150078, Abcam). Sections were counterstained with DAPI (cat no. D9542-5MG, Sigma-Aldrich, Darmstadt, DE) and mounted with Fluor-shield histology mounting medium (Catalog F6182-10MG, Sigma-Aldrich). Slides were examined using a Nikon Eclipse Ni microscope equipped with filters appropriate for the fluorochrome to be analyzed. Images were recorded with a Nikon DS-Qi1Nc digital camera and NIS Elements software BR 4.20.01. Confocal microscopy determinations were performed at ×40 magnification on at least 120 CD42b positive cells (MKs) per area (0.720 mm^2^) of bone marrow section per mouse. We have analyzed a total of 6 mice treated with vehicle, three mice treated with Reparixin for three days and 7 mice treated with Reparixin for 37 days.

### Data Analysis

Data were analyzed and plotted using GraphPad Prism 8.0.2 software (GraphPad Software, San Diego, California United States) and presented as Mean (± SD) or as box charts, when appropriate. Shapiro–Wilk test was used to confirm that the data are normally distributed. Comparisons between the two groups were performed with one way ANOVA while comparisons between multiple groups were performed with the Tukey’s multiple comparisons test. Linear correlations were calculated by the Pearson *R* test. Differences were considered statistically significant with a p <0.05.

## Results

### Treatment With Reparixin Is Well Tolerated Without Significant Changes in Body Weight and Blood Counts

**Figure 2:**
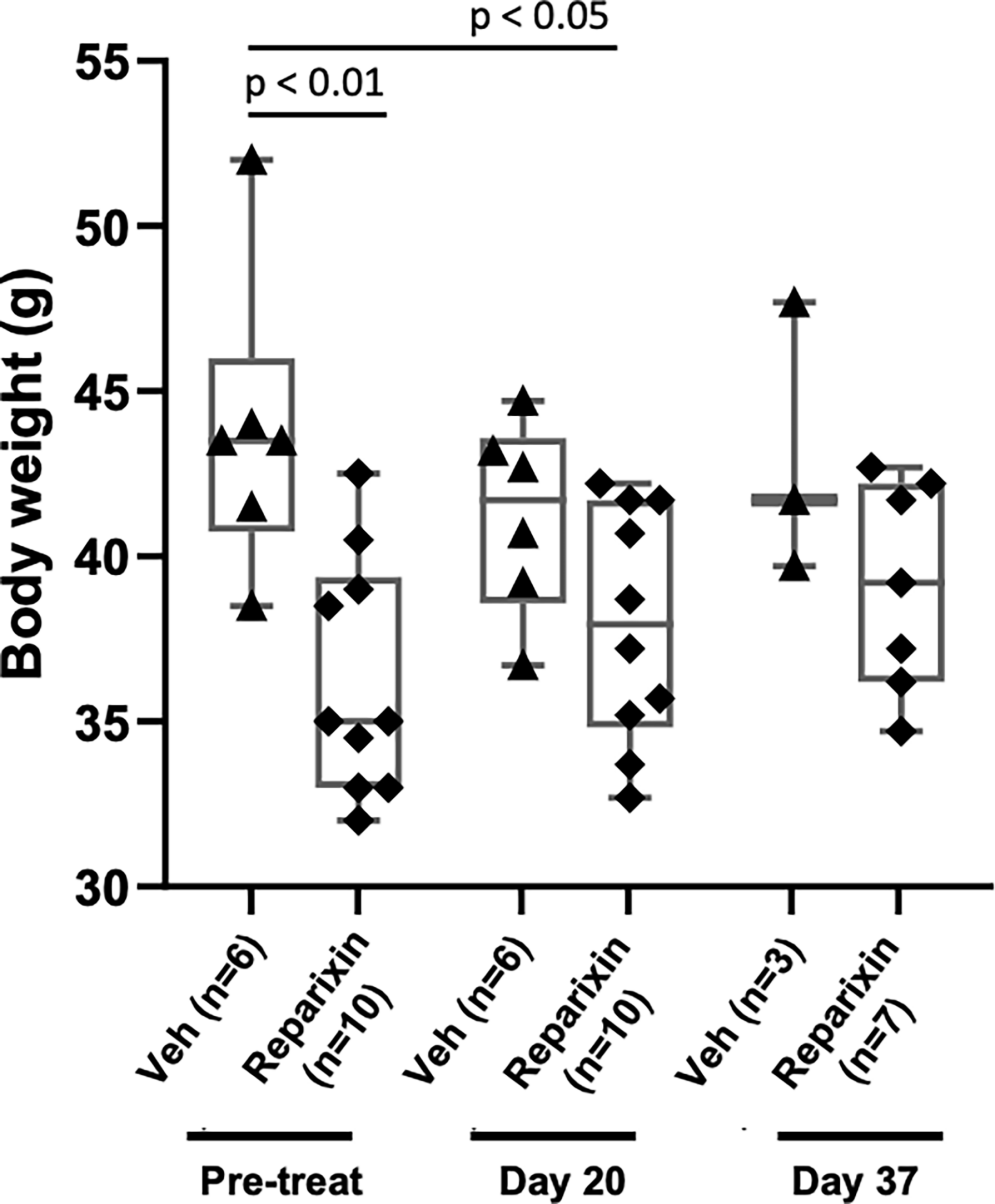
Treatment with Reparixin did not affect animal weights. Body weight determinations performed before (Pre-treat) and after 20 or 37 days of treatment with Reparixin. Data are presented as box charts and as value per individual mouse (each symbol a different mouse). The data are normally distributed by Shapiro–Wilk test and the Reparixin pre-treatment (p < 0.01) and at day 20 (p < 0.05) group are statistically different from the vehicle group by Turkey’s multiple comparison test.

The body weight of the mice included in the two experiments and its variations during treatment are shown in **Figure 2**. Probably due to sampling bias, before treatment the weight of the mice included in the vehicle and Reparixin group is statistically different (p <0.01). The difference in weight of the Reparixin-treated mice remains statistically different from that of the vehicle mice at day 20 (p <0.05). However, there is no statistically significant difference between the Reparixin-treated mice and the corresponding vehicle group both at days 20 and 37 (**Figure 2**). The wellbeing of the treated mice was monitored daily by a veterinarian. Death was not recorded and the mice remained active with no significant changes in behavior (no lethargy, no excessive grooming, no change in coat luster) during all the period of observation. The Hct and WBC counts remained within normal ranges in all experimental groups, while Plt counts were low as has been previously observed in *Gata1* ^low^ mice (**Table 1**). In-depth analyses of the WBC populations, revealed significant greater neutrophil counts at day 37 versus day 20 (**Table 1**), while the lymphocyte and monocyte counts remained similar among groups (**Figure S1**).

**Table 1:** Blood parameters observed in mice treated with vehicle or with Reparixin for 20 or 37 days.

	Htc(%)	WBC (×10^3^/μL)	Neutrophils (×10^3^/μL)	Plt (×10^3^/μL)
**Vehicle** (n = 5)	34.32 ± 3.87	2.78 ± 0.55	0.35 ± 0.32	187.80 ± 26.12
**Reparixin day 20** (n = 3)	35.63 ± 3.45	3.27 ± 0.72	0.30 ± 0.18	181.30 ± 53.30
**p-values** (versus vehicle)	0.8771	0.8103	0.9868	0.9862
**Reparixin day 37** (n = 6)	30.92 ± 3.58	3.57 ± 1.43	1.02 ± 0.52	99.83 ± 71.92
**p-values** (versus vehicle)	0.3136	0.4684	0.0465	0.0591
**p-values** (Versus Reparixin day 20)	0.2082	0.9173	0.0691	0.1429

Data are presented as Mean (± SD) and p-values are calculated by Tukey’s multiple comparisons test. Hct, hematocrit; Ptl, platelet count; WBC, white blood cell count. The number of mice included in each experimental group is indicated by n.

### Plasma Levels of Reparixin

**Figure 3:**
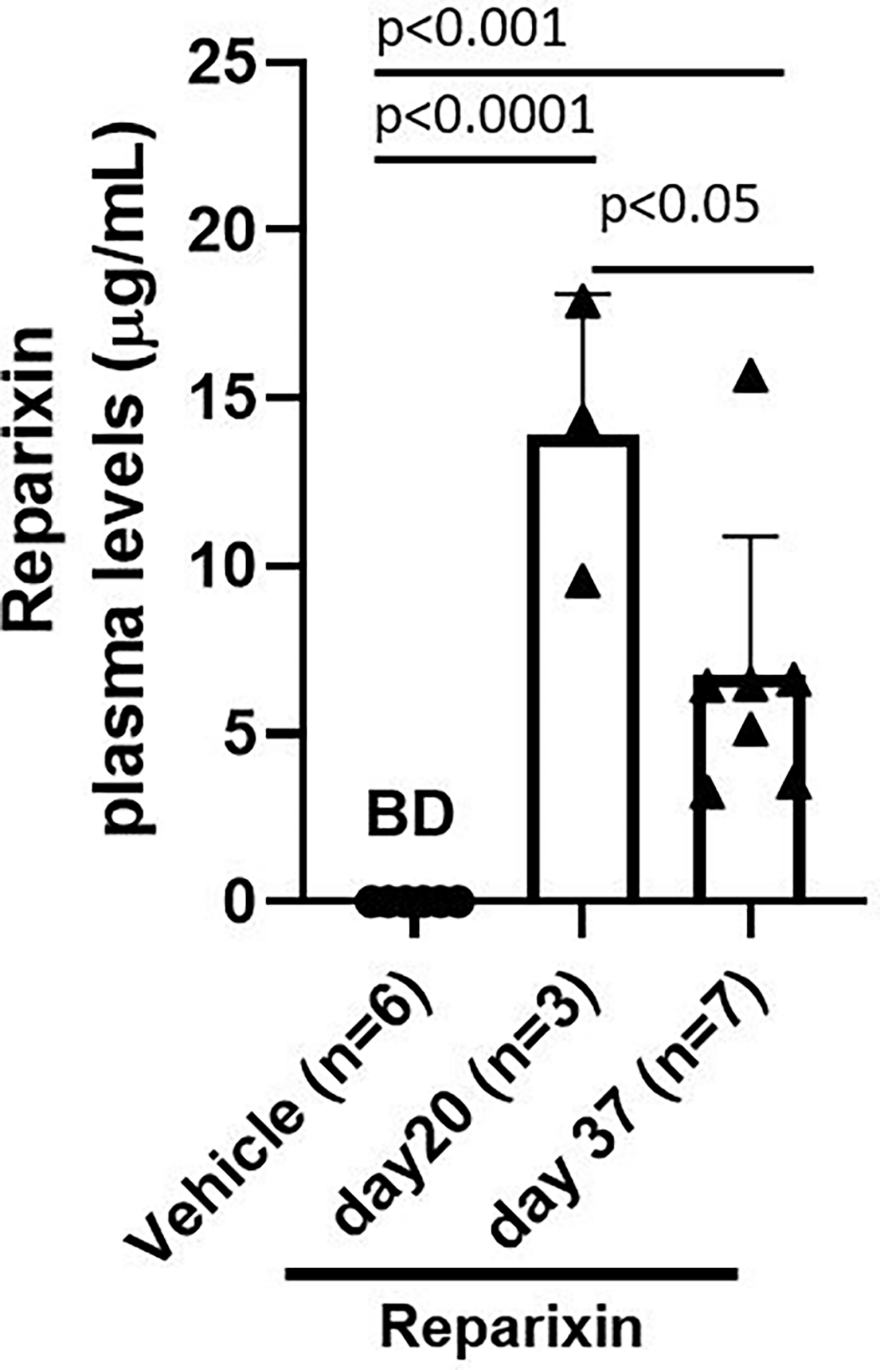
Plasma levels (μg/ml) of Reparixin detected at days 20 and 37. Plasma levels of Reparixin were significantly higher in *Gata1* ^low^ mice scarified at day 20 as compared to those sacrificed at day 37. Data are presented as Mean (± SD) and are analyzed by Tukey’s multiple comparisons test. Dots represent values observed in the individual mice. P < 0.05 was considered statistically significant. Abbreviations: BD, below detectable levels (i.e., <20% of the lower limit of quantitation (0.2 µg/ml).

According to the specification provided by the manufacturer, Alzet model 2002 mini-pumps have an approximate 0.2 ml reservoir that delivers a preloaded drug or vehicle solutions continuously for at least 14 days at a rate of 5 μl/h. To confirm that mice remained to the drug at the time of sacrifice, the plasma levels of Reparixin at days 20 and 37 were determined. Plasma levels ranged from 3.24 to 17.87 μg/ml. These levels are similar to those observed in previous experiments using the same device (36). Notably, plasma levels of Reparixin were significantly higher in mice treated for 20 days (13.90 ± 4.18) compared to those that underwent a second implantation and were treated for 17 additional days (6.71 ± 4.18) (**Figure 3**). Although the levels of drug detected at the second time-point were similar to those determined in prior studies (36), the observation that mice treated with the same concentration for a longer time have a plasma level of the drug lower than those treated for lower times is puzzling. It is possible that changes in the cell composition of the longer treated mice lead to greater amounts of tissue-bound Reparixin reducing the free levels of the drug found in plasma. However, it is also possible that the plasma levels of Reparixin at the two time points reflect differences in the efficiency of the derma to absorb the drug. This alternative hypothesis is supported by the observation that, since GATA1 regulates the differentiation of dermal mast cells, derma of *Gata1^low^* mice contains great numbers of these cells (37). In addition, wild-type CD1 mice, the background in which we harbor the *Gata1^low^* mutations express a systemic pro-inflammatory signature which determines chronic dermatitis with dermal fibrosis (32). This baseline dermal fibrosis is also present in *Gata1^low^* mice (ARM, unpublished observations) and it is possibly exacerbated once the mast cells are activated by the mechanical stress induced by the mini-pumps implanted subcutaneously, reducing the efficiency of the dermal absorption and of the plasma levels of the drug. Therefore, the plasma levels of Reparixin are a true reflection of the concentration of the drug delivered by the mini-pumps to the animals.

### The Reduction of Fibrosis in the BM of *Gata1* ^low^ Mice Induced by Reparixin Correlates With the Concentration of the Drug in the Blood

The BM from *Gata1* ^low^ mice is characterized by reduced cellularity due to accumulation of reticulin fibers with age (30). Overall, the treatment had modest effects on BM cellularity (**Table 2**).

**Table 2:** Bone marrow cellularity and spleen weight determined in *Gata1* ^low^ mice treated either with vehicle or with Reparixin for 20 or 37 days.

	Cells/femur (×10^6^)	Spleen weight (g)
**Vehicle** (n = 6)	20.55 ± 5.83	0.33 ± 0.13
**Reparixin day 20** (n = 3)	22.24 ± 0.85 (p = 0.91)	0.26 ± 0.05 (p = 0.51)
**Reparixin day 37** (n = 7)	21.68 ± 6.49 (p = 0.93)	0.35 ± 0.06 (p = 0.93)

Data are presented as Mean (± SD) and p-values were calculated with respect to vehicles by Tukey’s multiple comparisons test.

**Figure 4:**
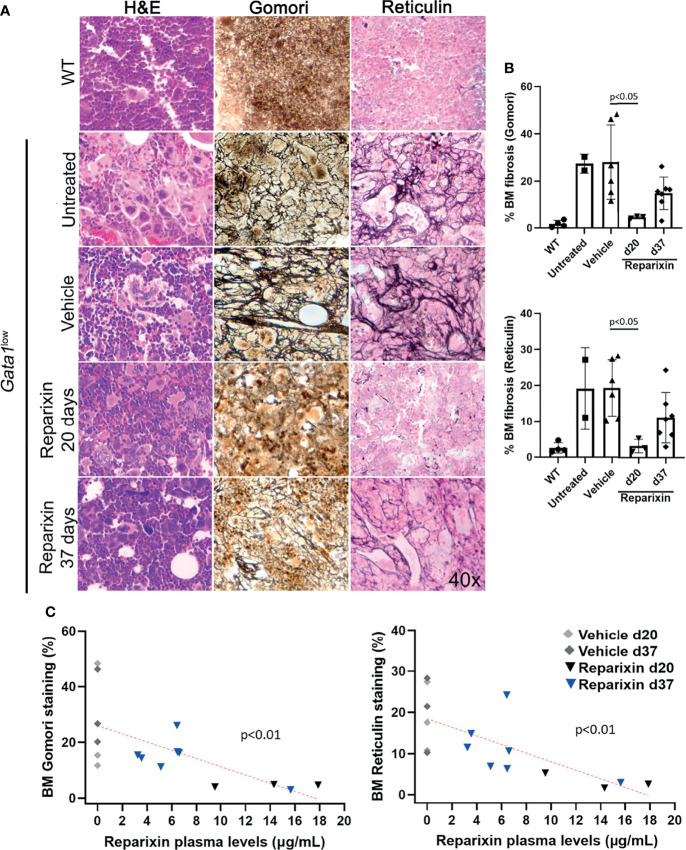
Reparixin decreases in a concentration-dependent fashion the fibrosis present in the BM from *Gata1* ^low^ mice. **(A)** H&E, Reticulin, and Gomori staining of BM sections from representative Gata1^low^ mice treated either with vehicle or Reparixin for 20 or 37 days, as indicated. Untreated *Gata1* ^low^ and WT littermates are presented as positive and negative controls, respectively. Magnification ×400. **(B)** Levels of fibrosis quantified by computer image analyses on BM sections stained with Gomori or Reticulin from multiple *Gata1* ^low^ mice treated either with vehicle or Reparixin, as indicated. Data are presented as Mean (± SD) and are analyzed by Tukey’s multiple comparisons test. P < 0.05 was considered statistically significant. **(C)** Linear regression analyses between fibrosis plasma concentration or Reparixin in individual mice (Pearson R = −0.66, p < 0.01 for Gomori staining; Pearson R = −0.71, p < 0.01 for Reticulin staining). Each dot represents an individual mouse.

However, a reduction in BM fibrosis was observed by both Gomori and Reticulin stainings in mice treated with Reparixin compared to those treated with vehicle alone to a statistically significant degree on day 20 (**Figures 4A, B**). Since mice treated for 20 days had higher concentrations of the drug in the plasma, we assessed whether the effects exerted by Reparixin on fibrosis was concentration-dependent rather than time-dependent by performing concentration/effect correlation analyses. Notably, this analysis revealed that the levels of fibrosis were inversely correlated with the plasma levels of the drug in individual mice (**Figure 4C**).

### The Reduction of Fibrosis in the Spleen of *Gata1* ^low^ Mice Induced by Reparixin Correlates With the Levels of the Drug in the Blood

**Figure 5:**
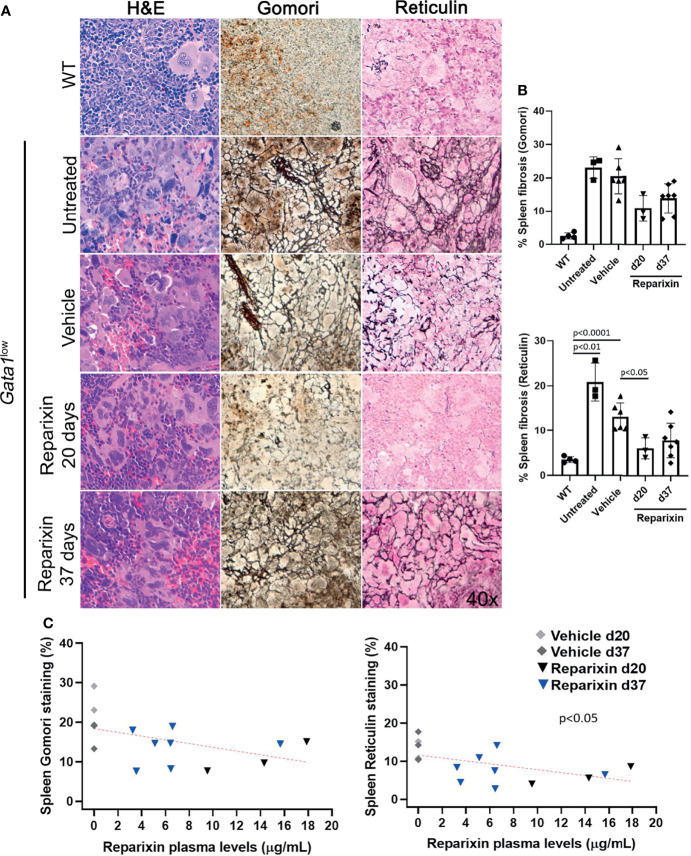
Reparixin decreased in a concentration-dependent fashion the degree of fibrosis present in the spleen of *Gata1* ^low^ mice. **(A)** H&E, Reticulin, Gomori and reticulin stainings of spleen sections from representative *Gata1^low^* mice treated either with vehicle or Reparixin for 20 or 37 days, as indicated. Untreated *Gata1* ^low^ and WT littermates are presented as positive and negative controls, respectively. Magnification ×400. **(B)** Levels of fibrosis quantified by image analyses in spleen sections stained with Gomori or Reticulin from *Gata1* ^low^ mice treated either with vehicle or Reparixin, as indicated. Data were presented as Mean (± SD) and were analyzed by Tukey’s multiple comparisons test. P < 0.05 was considered statistically significant. **(C)** Linear regression analyses of fibrosis and plasma levels of Reparixin in individual mice (Pearson R = −0.48, not significant for Gomori staining; Pearson R = −0.53, p < 0.05 for Reticulin staining). Each dot represents a single mouse.

*Gata1* ^low^ mice developed extramedullary hematopoiesis with fibrosis in the spleen and associated splenomegaly. Despite the fact that the treatment with Reparixin did not induce significant changes in spleen volumes (**Table 2**), histological analyses indicated a remarkable reduction in the fibrosis expressed by the spleen in the Reparixin-treated mice (**Figure 5A**). As for BM, the reductions observed in the spleen were statistically significant only at day 20 (**Figure 5B**) but analyses of all the time points revealed a significant inverse correlation between fibrosis detected by reticulin staining and plasma levels of Reparixin in individual mice (**Figure 5C**).

### Treatment With Reparixin Reduced the Levels of TGF-β1, But Not That of mCXCL1or its Receptors CXCR1/2 in the BM of *Gata1* ^low^ Mice

**Figure 6:**
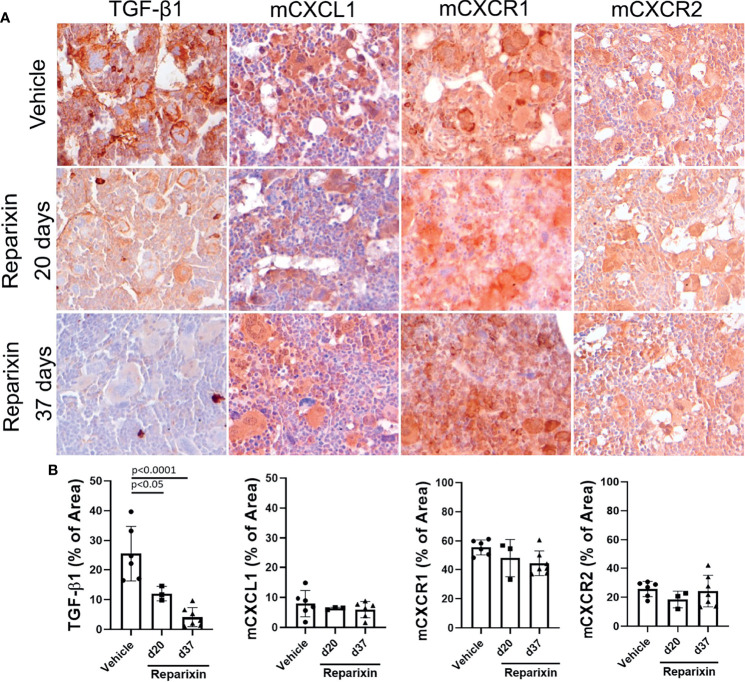
Treatment with Reparixin decreases the TGF-β1 content of the BM from *Gata1* ^low^ mice. **(A)** Immunohistochemical staining for TGF-β1, mCXCL1, mCXCR1, and mCXCR2 of BM sections from representative *Gata1* ^low^ mice treated either with vehicle or Reparixin for 20 or 37 days, as indicated. Magnifications ×40. **(B)** Quantification by computer assisted imaging of the TGF-β1, mCXCL1, mCXCR1, and mCXCR2 content in the BM from Gata1low mice treated with either with vehicle or Reparixin for 20 or 37 days, as indicated. Data are presented as Mean (± SD) and were analyzed by Tukey’s multiple comparisons test. Values observed in individual mice are presented as dots. P < 0.05 were considered statistically significant.

To gain greater insights on the effects of Reparixin on fibrosis reduction in *Gata1^low^* mice, we determined by immunohistochemistry the content of CXCL1, and of its receptors CXCR1 and CXCR2, in the BM of *Gata1low* mice treated either with Reparixin or with vehicle. We had previously demonstrated that the BM from *Gata1* ^low^ mice contain high levels of TGF-β and express an altered TGF-β signature which was thought to be the cause of the marrow fibrosis since treatment of the mice with the receptor-1(R1) kinase inhibitor SB431542 (31) or with a TGF-β trap (38) reversed both the abnormal TGF-β signature and the increase in marrow fibrosis, restoring hematopoiesis in BM, of these mice. On the basis of these data, the content of TGF-β in the BM of *Gata1low* mice treated either with Reparixin or with vehicle was determined. These analyses indicated that the MKs from the Reparixin-treated mice express significantly lower levels of TGF-β1 than the corresponding cells from animals receiving vehicle alone. By contrast the levels of CXCL1, CXCR1, and CXCR2 expressed by the Reparixin-treated MKs remained comparable to that of the vehicle-treated cells (**Figure 6**). Although the reductions in TGF-β1 content were not correlated with the plasma levels of Reparixin of individual mice (data not shown), these results suggest that Reparixin may decrease fibrosis by reducing the levels of TGF-β1.

### Reparixin Increases the GATA1 Content While Reducing That of Collagen III in MKs From the Bone Marrow of *Gata1* ^low^ Mice

Previous studies have established that in both MF patients and in MPN driver mutation animal models of myelofibrosis the reduced levels of GATA1 is due to a ribosomopathy that reduces the GATA1 content in the malignant MKs (2, 39). In addition, MK-restricted expression of *JAK2*V617F, the most common driver mutation of MF, is sufficient to induce myelofibrosis in mice (40). The finding from the Balduini laboratory that a large proportion of the malignant MKs in the BM of MF patients express collagen, suggests that hypomorphic GATA1 MKs are directly responsible for the fibrosis observed in these patients (41). This hypothesis is supported by recent single cell profiling of BM cells that has identified a previously unrecognized population of MKs poised to exert niche-functions by secreting collagen and other extracellular matrix proteins. These niche supporting MKS morphologically resemble immature MKs (low ploidy levels with reduced presence of granules and platelet-territories in their cytoplasm) the maturation of which is sustained by low levels of GATA1 and high levels of TGF-β signaling (42–44).

**Figure 7:**
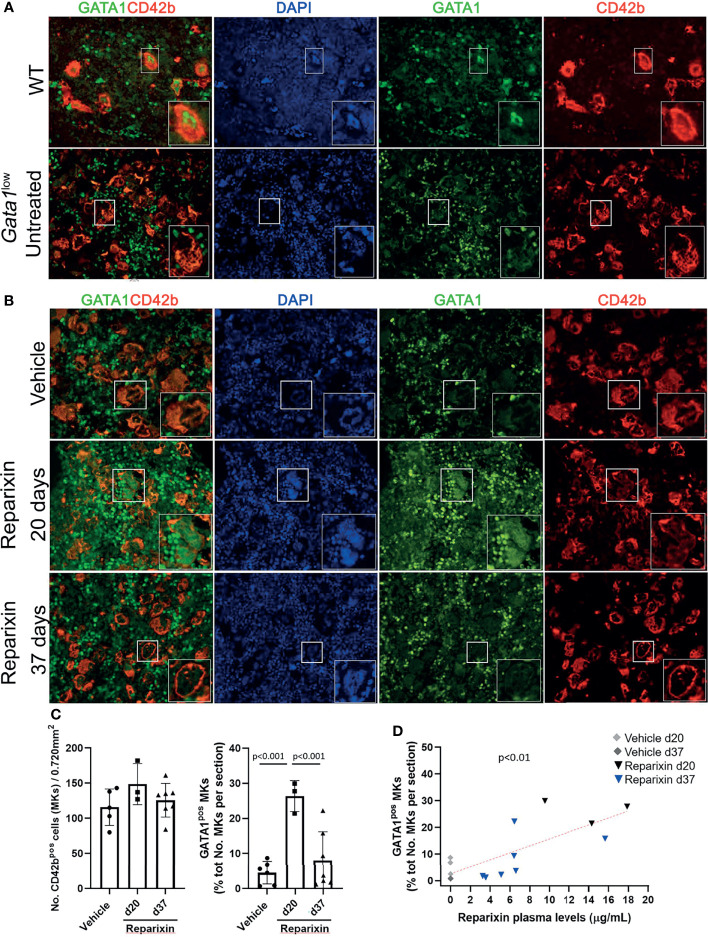
Reparixin restores in a concentration-dependent fashion the GATA1 content of the MKs in the BM of *Gata1^low^* mice. **(A)** Double immunofluorescence staining with GATA1 (FITCH-green) and CD42b (TRITCH-red), as a marker of MKs (45), antibodies of BM sections from representative wild-type (WT) and untreated *Gata1* ^low^ mice, as indicated. The hypomorphic *Gata1* ^low^ mutation selectively reduces expression of GATA1 in the MKs from the BM of *Gata1* ^low^ mice compared to the correspondent cells from WT littermates. Magnification ×400. **(B)** Double immunofluorescence staining with GATA1 (FITCH-green) and CD42b (TRITCH-red) of BM sections from representative mice treated either with vehicle or Reparixin for 20 or 37 days, as indicated. Magnification ×400. The boxes in **(A)** and **(B)**_ indicate the MKs shown at greater magnification on the bottom of the panels. **(C)** Frequency of MKs and percentage of MKs positive for GATA1 in BM sections from multiple treated mice. Data are presented as Mean (± SD) and as values in individual mice (each symbol a mouse). Results were analyzed by Tukey’s multiple comparisons test. P < 0.05 were considered statistically significant. **(D)** Correlation between the percent of MKs positive for GATA1and plasma levels of the Reparixin in individual mice (Pearson R = 0.76, p < 0.01). Each dot represents a single mouse.

Since the hypomorphic *Gata1* ^low^ mutation is characterized by reduced expression of GATA1 in MKs and the BM from these mice express high levels of TGF-β (32, 44), we hypothesized that this fibrosis may be sustained by increased numbers of niche-poised MKs with low levels of GATA1 due to the increase in BM TGF-β and that the reduced levels of TGF-β induced by Reparixin would reduce the degree of marrow fibrosis by reducing the frequency of this niche-poised MKs present in BM. To test this hypothesis, we first analyzed the content of GATA1 in MKs from BM sections of mice treated either with vehicle or with Reparixin (**Figure 7**). This analysis confirmed that the BM from *Gata1^low^* mice contained greater numbers of MKs and that the GATA1 content of these MKs was reduced (**Figure 7A**). Although the number of MKs in both vehicle- and Reparixin-treated mice remained greater than normal, the GATA1 content of the MKs from the Reparixin-treated mice was greatly increased (**Figures 7B, C**). Of note, the percentage of MKs expressing high GATA1 levels was directly correlated with the plasma levels of Reparixin detected in individual mice (**Figure 7D**).

**Figure 8:**
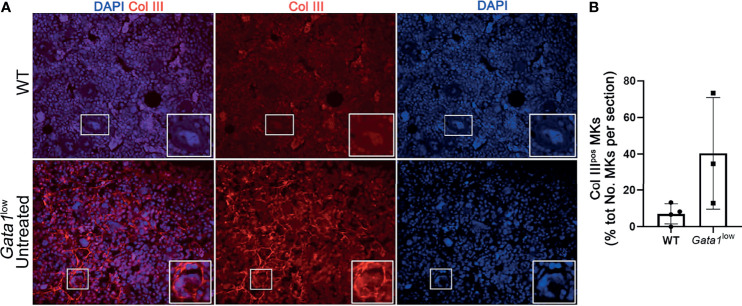
The bone marrow from Gata1^low^ mice contains great numbers of megakaryocytes that express Collagen III. **(A)** Representative immunofluorescence staining with an anti-Col III (TRITCH-red) antibody showing that the MKs from *Gata1* ^low^ mice express higher levels of Col III than those from their WT littermates. Megakaryocytes were recognized on the basis of their morphology (size and morphology of the nucleus). Magnification ×400. The white boxes indicate representative MKs shown at greater magnification on the right bottom side of each panel. **(B)** Percent of MKs positive for CollIII (Col III^pos^ MKs) over the total numbers of MKs observed in BM sections from WT and *Gata1* ^low^ littermates. Data are presented as Mean (± SD) and as frequency per individual mouse (dots) and were analyzed by One-way ANOVA. Each dot represents a single mouse. P < 0.05 was considered statistically significant.

We then compared the percent of MKs expressing Collagen III in the BM of *Gata1* ^low^ and WT mice (**Figures 8A, B**). Since the anti-ColIII antibody is conjugated with the same fluorochrome that labels most of the commercially available mouse CD42 antibodies, in these studies MKs were recognized on the basis of their large size and their polylobulated nuclei as revealed by DAPI staining. Greater number of the MKs in the BM from *Gata1^low^* expressed increased levels of collagen III while these cells were rare in WT mice, suggesting that the BM from *Gata1^low^* mice was enriched for niche-poised MKs.

**Figure 9:**
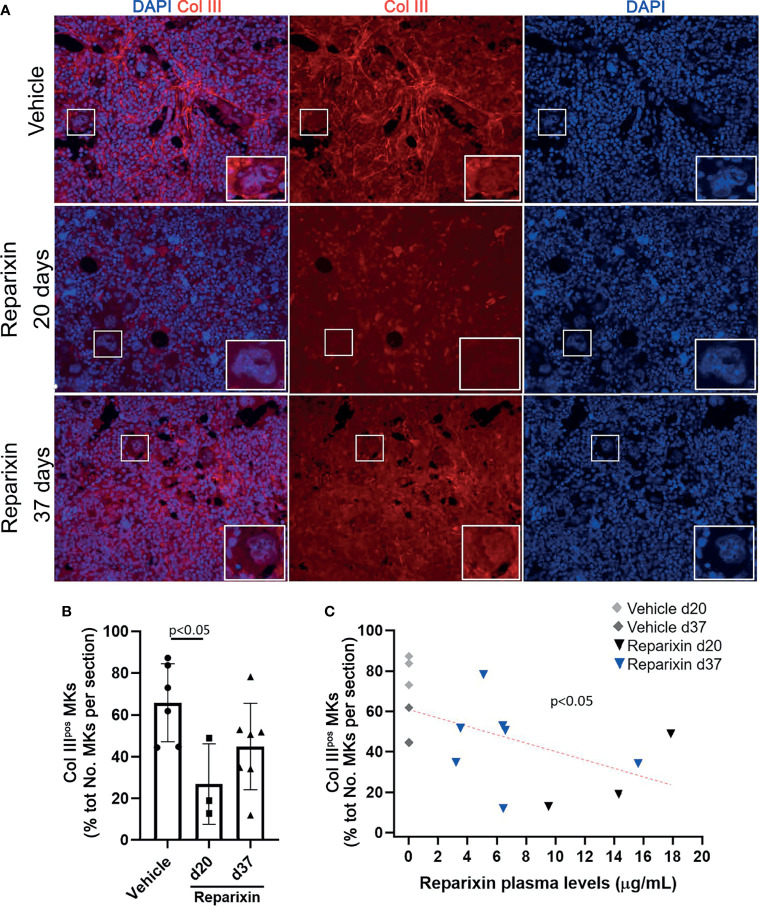
Reparixin reduces in a concentration-dependent fashion the frequency of Col III^pos^ MKs in the bone marrow of *Gata1* ^low^ mice. **(A)** Immunofluorescence analyses with an anti-Col III (TRITCH-red) antibody of BM sections from representative *Gata1* ^low^ mice treated either with vehicle or with Reparixin for 20 or 37 days, as indicated. Megakaryocytes were recognized by morphology. Magnification ×400. The white rectangles indicate representative MKs shown at greater magnification on the bottom right of each panel. **(B)** Frequency of MKs positive for Col III over the total number of MKs per sections quantified by computer image analyses. Data are presented as Mean (± SD) and as values per single mouse (each dot a mouse) and were analyzed by Tukey’s test multiple comparisons test. P < 0.05 was considered statistically significant. **(C)** Linear regression analyses between the frequency of CollIII^pos^ MKs and plasma levels of Reparixin in individual mice (Pearson R = −0.53, p < 0.05). Each dot represents a single mouse.

Notably, we then demonstrated that treatment with Reparixin reduced in a concentration-dependent fashion the frequency of MKs expressing collagen III in the BM from *Gata1* ^low^ mice while the frequency of these cells in the BM of the vehicle treated mice remained increased (**Figure 9**).

### Niche-Poised and Platelet-Poised MK Have Distinctive Morphologies

**Figure 10:**
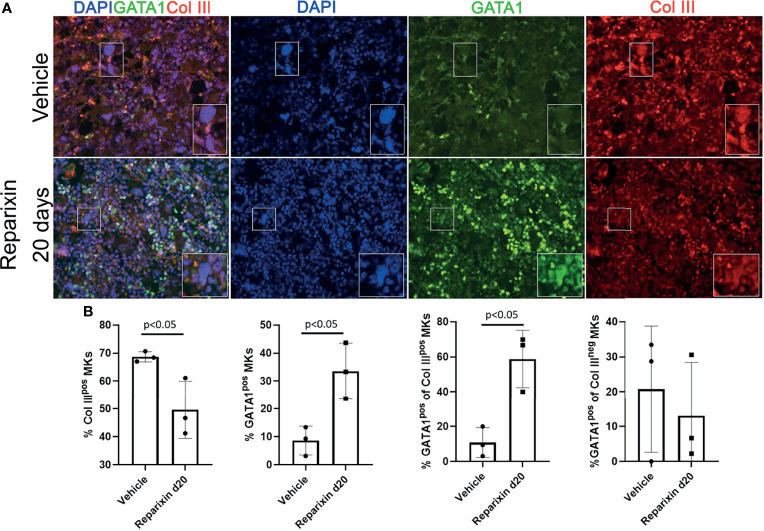
Reparixin increases the GATA1 content in the MK subpopulation that express high levels of Col III. **(A)** Double immunofluorescence staining with anti-GATA1 (FITCH-green) and Col III (TRITCH-red) antibodies of BM sections from representative *Gata1* ^low^ mice treated with either vehicle or Reparixin for 20 days. Magnification ×400. The rectangles indicate a representative MK shown at greater magnification on the bottom right of each panel. **(B)** Frequency of MKs positive for Col III (Col III^pos^ MKs), for GATA1 (Gata1^pos^ MKs) and of the subsets of MKs positive or negative for Col III that were also positive for GATA1, respectively. Data are presented as Mean (± SD) and as values for individual mouse (each mouse a dot). Statistical analyses were performed by One-way ANOVA. P < 0.05 was considered statistically significant.

On average, niche-poised MKs are smaller than platelet-poised cells and their nuclei contains lower number of lobi (43). The analyses of the morphology of the MKs shown in **Figure 7** at greater magnification revealed that the MKs from the Reparixin-treated mice that contained GATA1 are on average smaller and with less lobated nuclei than those that were negative for GATA1 (**Figure S2**). These results suggest that Reparixin may have specifically increased the GATA1 content in the niche-poised MKs, explaining why, in spite of the increase of GATA1 in the MKs, the Plt numbers of the treated mice did not increase (**Table 1**). This hypothesis was tested by performing immunofluorescence studies to assess whether Reparixin increased the GATA1 content in the same MK population that expresses collagen (**Figure 10**). This analysis was performed only on the mice that had been treated for day 20 which exhibited the greatest reduction of fibrosis. The frequency of MKs expressing collagen III which were also positive for GATA1 was significantly greater in mice treated with Reparixin suggesting that Reparixin had specifically increased GATA1 content in niche-poised MKs, possibly hampering their pro-fibrotic functions.

## Discussion

hCXCL8 is one of the pro-inflammatory cytokines expressed at high levels in MF and is associated with the poorest patient outcomes (3). In addition, malignant CD34^+^ cells and MKs from MF patients express high levels of hCXCL8 (10). We have now determined that the BM from the *Gata1* ^low^ MF mouse model contains MKs that not only express increased levels of TGF-β, but also of mCXCL1, the murine equivalent of hCXCL8, and that the MKs from these mice express higher levels of CXCR1 and CXCR2 receptors. The recent consideration of CXCR1/R2 allosteric inhibitors for therapy of inflammatory lung diseases associated with fibrosis (26, 27) suggests that these inhibitors might be effective in treating BM fibrosis. This hypothesis was tested in the present study that assessed the effects of the CXCR1/R2 inhibitor Reparixin on the myelofibrotic phenotype expressed by *Gata1* ^low^ mice.

In accordance with the observations that allosteric modulation of CXCR1/2 is well tolerated (46–48), treatment with Reparixin showed no significant changes in body weight and in blood parameters in *Gata1^low^* mice, and no deaths were recorded during the entire period of observation.

Having found that an intrinsic variability in circulating drug levels was associated with prolonged administration of Reparixin by mini-pumps (36 and this manuscript), we performed correlative analyses to assess concentration-dependent treatment effects. Notably, by histological analysis we observed that Reparixin reduced in a concentration-dependent fashion the degree of BM and splenic fibrosis of *Gata1* ^low^ mice. Since CXCL1 and its CXCR1 and CXCR2 receptors contribute to the control of megakaryocytic proliferation, differentiation, and ploidy in MF (9), we evaluated whether inhibition of the CXCR1/R2 signaling might reduce the fibrosis by restoring the MKs abnormalities observed in *Gata1* ^low^ mice.

First, immunohistochemistry staining showed for the first time that mCXCR1, the recently characterized murine orthologue of hCXCR1 is highly expressed, together with mCXCR2 specifically in the MKs from *Gata1* ^low^ mice. Consistent with the fact that allosteric modulation does not block the binding of the endogenous ligand to its receptors or alter its constitutive activity (22), we observed that Reparixin treatment did not alter the expression of CXCL1 or CXCR1/R2 receptors by MKs from the BM of *Gata1* ^low^ mice. Since CXCR1/CXCR2 are also activated by CXCL6 and MIP2, we may not formally exclude that altered levels of these two chemokines are also involved in the development of myelofibrosis in our model and that their levels where normalized by treatment with Reparixin. This hypothesis will be tested as part of a separate study.

We observed, however, that the BM of Reparixin-treated mice expressed lower levels of TGF-β as compared to vehicle treated mice. The effect of Reparixin on TGF-β expression was independent of the plasma concentration of Reparixin. Since Reparixin is a >100 fold more potent inhibitor of CXCR1 (both h and m) than CXCR2 (both h and m), (22, 23), we hypothesize that TGF-β production by murine MKs is primarily regulated by mCXCR1 and therefore is not influenced by the variability in circulating drug levels. The suggested role of mCXCR1 in the regulation of TGF-β content is supported by recent data highlighting the prominent role of the CXCL6/CXCR1 axis in the release of TGF-β by liver Kupffer cells both in patients with liver fibrosis and in a mouse model of carbon tetrachloride-induced chronic liver injury and fibrosis (49). We may not exclude however, that Reparixin reduced TGF-β content in the bone marrow indirectly by reducing the frequency of pathological MKs emperipolesis with the neutrophils which triggers the release of this factor in the bone marrow. In fact, since it is well known that CXCL1 induces neutrophil chemotaxis (50), it is possible that Reparixin, by decreasing neutrophil chemotaxis toward the MKs reduces their pathological emperipolesis with these cells reducing the amount of TGF-β they release in the microenvironment. This hypothesis is currently under investigation as part of a separate study (Dunbar et al., under revision). In conclusion, our results indicate that, in addition to CXCR2 indicated by loss of function studies in mouse models, (Dunbar et al., under review), CXCR1 is also involved in the regulation of profibrotic signaling at least in *Gata1* ^low^ mice.

We previously reported that pharmacological inhibition of TGF-β signaling in *Gata1* ^low^ mice restored the maturation of MKs and increased their content of GATA1 (31). Since we observed a significant decrease of the TGF-β expressed by MKs following Reparixin-treatment, we hypothesized that Reparixin might restore MK functions by reducing TGF-β content. This hypothesis is counterintuitive since Reparixin did not increase platelets numbers, which are typically low in *Gata1* ^low^ mice. This apparent paradox was explained by recent single cell profiling analyses indicating that murine BM contains three distinctive MK populations, each one exerting a different function. In addition to MKs poised to generate platelets, the BM contains MK poised to exert immune functions in the lungs (51) or niche functions during the embryogenesis but possibly also in adult organs undergoing tissue repair (42–44, 51). The BMs from *Gata1* ^low^ mice, and also that of MF patients, are characterized by an increased proportion of immature MKs that express increased levels of collagen (41, 52 and this manuscript). By immunostaining, we observed that Reparixin increased GATA1 expression while reducing the collagen III content in *Gata1* ^low^ mouse MKs. Notably, we demonstrated that the increase of GATA1 levels was most evident in the subpopulation of MKs expressing collagen, suggesting that Reparixin may target the niche-poised MKs reducing their ability to mediate collagen deposition and fibrosis in this animal model. At the moment, there is little experimental indication on the mechanism(s) that increased GATA1 content in the MKs from the Reparixin-treated mice. On the basis of published data, we suggest that this increase is mediated by the decreased TGF-β levels observed in these mice. In fact, it is well known that TGF-β, through a mechanism still poorly identified, retains MK immature (53). Since MK maturation requires GATA1 upregulation [with consequently downregulation of GATA2, a maturation mechanism defined the GATA1 to GATA2 switch (54)], it is conceivable that TGF-β reduces MK maturation by reducing expression of GATA1 and increasing that of GATA2. This hypothesis has been directly tested by the finding that a small TGF-β inhibitor rescues the abnormal maturation (and myelofibrosis) in *Gata1^low^* mice by increasing the levels of *Gata1* mRNA while reducing those of *Gata2* (31, 55). Due to the hypomorphic mutation, *Gata1^low^* cells lacks one of the three major hypersensitive sites of the gene but still contain two other important regulatory sequences (56, 57), which may be the target of the TGF-β inhibitors which have been demonstrated to be capable to upregulate its expression (31). Although drugs that increase GATA1 in MKs, such as Aurora kinase inhibitors, have been shown to be effective in reduce fibrosis in myelofibrosis patients (58), it may be debated whether drugs, such as Reparixin, that increase GATA1 protein in a mouse model in which the transcription of the gene is reduced by deletion of its regulatory sequence, will be effective in patients where the GATA1 content is reduced by inefficient translation of GATA1 mRNA (39). However, since Reparixin appears to act *via* TGF-β and the TGF-β TRAP AVID200 is capable to downregulate GATA2 (and therefore presumably to upregulates GATA1), restoring the maturation of MKs expanded *in vitro* from CD34+ cells in the subset of patients who are responsive to the drug (38), we believe that Reparixin will be effective also in patients.

In conclusion, these results indicate that treatment with Reparixin rescues the MF phenotype of *Gata1^low^* mice and provides a rationale for considering Reparixin as a therapeutic option to treat MF patients.

## Data Availability Statement

The original contributions presented in the study are included in the article/**Supplementary Material**. Further inquiries can be directed to the corresponding authors.

## Ethics Statement

The animal study was reviewed and approved by the Institutional animal care committee according to the European Directive 86/609/EEC.

## Author Contributions

PV, FG, FM, MTM, LB and CG performed experiments and analyzed the data. PV and FG performed statistical analyses. GS reviewed all the histopathogical determinations. ARM and MA designed the study, interpreted the data and wrote the manuscript. All the authors read the manuscript and concur with its content. All authors listed have made a substantial, direct, and intellectual contribution to the work and approved it for publication.

## Funding

This study was supported by grants from the National Cancer Institute (P01-CA108671), the National Heart, Lung and Blood Institute (1R01-HL134684), the Associazione Italiana Ricerca Cancro (AIRC, IG23525), by funds from the Italian Ministry of Economic Development DM 02/08/2019 and DD 02/10/2019 grant proposal number 1410, and by the Dompé Farmaceutici Spa R&D.

## Conflict of Interest

MTM, LB, CG and MA are employees of Dompè Farmaceutici SPA and ARM received research funds from Dompè Farmaceutici SPA.

The authors declare that the research was conducted in the absence of any commercial or financial relationships that could be construed as a potential conflict of interest.

The authors declare that this study received funding from Dompè Farmaceutici SpA. The funder had the following involvement with the study: writing of the article and interpretation of the data.

## Publisher’s Note

All claims expressed in this article are solely those of the authors and do not necessarily represent those of their affiliated organizations, or those of the publisher, the editors and the reviewers. Any product that may be evaluated in this article, or claim that may be made by its manufacturer, is not guaranteed or endorsed by the publisher.

## References

[1] Tefferi A. Pathogenesis of Myelofibrosis With Myeloid Metaplasia. J Clin Oncol (2005) 23(33):8520–30. doi: 10.1200/JCO.2004.00.9316 16293880

[2] Vannucchi AM Pancrazzi A Guglielmelli P Di Lollo S Bogani C Baroni G. Abnormalities of GATA-1 in Megakaryocytes From Patients With Idiopathic Myelofibrosis. Am J Pathol (2005) 167(3):849–58. doi: 10.1016/S0002-9440(10)62056-1 PMC1698737 16127162

[3] Tefferi A Vaidya R Caramazza D Finke C Lasho T Pardanani A. Circulating Interleukin (IL)-8, IL-2r, IL-12, and IL-15 Levels Are Independently Prognostic in Primary Myelofibrosis: A Comprehensive Cytokine Profiling Study. J Clin Oncol (2011) 29(10):1356–63. doi: 10.1200/JCO.2010.32.9490 21300928

[4] Verstovsek S Mesa RA Gotlib J Gupta V DiPersio J Catalano JV. Long-Term Treatment With Ruxolitinib for Patients With Myelofibrosis: 5-Year Update From the Randomized, Double-Blind, Placebo-Controlled, Phase 3 COMFORT-I Trial. J Hematol Oncol (2017) 10(1):55. doi: 10.1186/s13045-017-0417-z 28228106 PMC5322633

[5] Harrison CN Vannucchi AM Kiladjian J-J Al-Ali HK Gisslinger H Knoops L. Long-Term Findings From COMFORT-II, A Phase 3 Study of Ruxolitinib vs Best Available Therapy for Myelofibrosis. Leukemia (2016) 30:1701. doi: 10.1038/leu.2016.323 27211272 PMC5399157

[6] Zimran A Durán G Giraldo P Rosenbaum H Giona F Petakov M. Long-Term Efficacy and Safety Results of Taliglucerase Alfa Through 5years in Adult Treatment-Naïve Patients With Gaucher Disease. Blood Cells Mol Dis (2019) 78:14–21. doi: 10.1016/j.bcmd.2016.07.002 27499018

[7] Russo RC Garcia CC Teixeira MM Amaral FA. The CXCL8/IL-8 Chemokine Family and its Receptors in Inflammatory Diseases. Expert Rev Clin Immunol (2014) 10(5):593–619. doi: 10.1586/1744666X.2014.894886 24678812

[8] Takeuchi K Higuchi T Yamashita T Koike K. Chemokine Production by Human Megakaryocytes Derived From CD34-Positive Cord Blood Cells. Cytokine (1999) 11:424–34. doi: 10.1006/cyto.1998.0455 10346982

[9] Emadi S Clay D Desterke C Guerton B Maquarre E Charpentier A. IL-8 and its CXCR1 and CXCR2 Receptors Participate in the Control of Megakaryocytic Proliferation, Differentiation, and Ploidy in Myeloid Metaplasia With Myelofibrosis. Blood (2005) 105:464. doi: 10.1182/blood-2003-12-4415 15454487

[10] Dunbar A Lu M Farina M Park Y Yang J Kim D. Increased Interleukin-8 (IL8)-CXCR2 Signaling Promotes Progression of Bone Marrow Fibrosis in Myeloproliferative Neoplasms. Blood (2020) 136(Supplement 1):6–7. doi: 10.1182/blood-2020-138843 32614958

[11] Murphy PM Tiffany HL. Cloning of Complementary DNA Encoding a Functional Human Interleukin-8 Receptor. Science (1991) 253:1280–3. doi: 10.1126/science.1891716 1891716

[12] Holmes WE Lee J Kuang WJ Rice GC Wood WI. Structure and Functional Expression of a Human Interleukin-8 Receptor. Science (1991) 253:1278–80. doi: 10.1126/science.1840701 1840701

[13] Wuyts A Van Osselaer N Haelens A Samson I Herdewijn P Ben-Baruch A. Characterization of Synthetic Human Granulocyte Chemotactic Protein 2: Usage of Chemokine Receptors CXCR1 and CXCR2 and *In Vivo* Inflammatory Properties. Biochemistry (1997) 36:2716–23. doi: 10.1021/bi961999z 9054580

[14] Bozic CR Gerard NP von Uexkull-Guldenband C Kolakowski LF Jr. Conklyn MJ Breslow R. The Murine Interleukin 8 Type B Receptor Homologue and its Ligands. Expression and Biological Characterization. J Biol Chem (1994) 269:29355–8. doi: 10.1016/S0021-9258(18)43882-3 7961909

[15] Cerretti DP Nelson N Kozlosky CJ Morrissey PJ Copeland NG Gilbert DJ. The Murine Homologue of the Human Interleukin-8 Receptor Type B Maps Near the Ity-Lsh-Bcg Disease Resistance Locus. Genomics (1993) 18:410–3. doi: 10.1006/geno.1993.1486 8288247

[16] Cacalano G Lee J Kikly K Ryan AM Pitts-Meek S Hultgren B. Neutrophil and B Cell Expansion in Mice That Lack the Murine IL-8 Receptor Homolog. Science (1994) 265:682–4. doi: 10.1126/science.8036519 8036519

[17] Lee J Cacalano G Camerato T Toy K Moore MW Wood WI. Chemokine Binding and Activities Mediated by the Mouse IL-8 Receptor. J Immunol (1995) 155:2158–64. 7636264

[18] Fu W Zhang Y Zhang J Chen WF. Cloning and Characterization of Mouse Homolog of the CXC Chemokine Receptor CXCR1. Cytokine (2005) 31:9–17. doi: 10.1016/j.cyto.2005.02.005 15967374

[19] Moepps B Nuesseler E Braun M Gierschik P. A Homolog of the Human Chemokine Receptor CXCR1 is Expressed in the Mouse. Mol Immunol (2006) 43:897–914. doi: 10.1016/j.molimm.2005.06.043 16084593

[20] Fan X Patera AC Pong-Kennedy A Deno G Gonsiorek W Manfra DJ. Murine CXCR1 is a Functional Receptor for GCP-2/CXCL6 and Interleukin-8/CXCL8. J Biol Chem (2007) 282(16):11658–66. doi: 10.1074/jbc.M607705200 17197447

[21] Wuyts A Haelens A Proost P Lenaerts JP Conings R Opdenakker G. Identification of Mouse Granulocyte Chemotactic Protein-2 From Fibroblasts and Epithelial Cells. Functional Comparison With Natural KC and Macrophage Inflammatory Protein-2. J Immunol (1996) 157:1736–43. 8759763

[22] Bertini R Allegretti M Bizzarri C Moriconi A Locati M Zampella G. Noncompetitive Allosteric Inhibitors of the Inflammatory Chemokine Receptors CXCR1 and CXCR2: Prevention of Reperfusion Injury. PNAS (2004) 101(32):11791–6. doi: 10.1073/pnas.0402090101 PMC511013 15282370

[23] Citro A Valle A Cantarelli E Mercalli A Pellegrini S Liberati D. CXCR1/2 Inhibition Blocks and Reverses Type 1 Diabetes in Mice. Diabetes (2015) 64(4):1329–40. doi: 10.2337/db14-0443 25315007

[24] Allegretti M Bertini R Bizzarri C Beccari A Mantovani A Locati M. Allosteric Inhibitors of Chemoattractant Receptors: Opportunities and Pitfalls. Trends Pharmacol Sci (2008) 29(6):280–6. doi: 10.1016/j.tips.2008.03.005 18423629

[25] Jurcevic S Humfrey C Uddin M Warrington S Larsson B Keen C. The Effect of a Selective CXCR2 Antagonist (AZD5069) on Human Blood Neutrophil Count and Innate Immune Functions. Br J Clin Pharmacol (2015) 80(6):1324–36. doi: 10.1111/bcp.12724 PMC4693488 26182832

[26] Cheng IY Liu CC Lin JH Hsu TW Hsu JW Li AF. Particulate Matter Increases the Severity of Bleomycin-Induced Pulmonary Fibrosis Through KC-Mediated Neutrophil Chemotaxis. Int J Mol Sci (2019) 21(1):227. doi: 10.3390/ijms21010227 PMC6981983 31905700

[27] Mattos MS Ferrero MR Kraemer L Lopes GAO Reis DC Cassali GD. CXCR1 and CXCR2 Inhibition by Ladarixin Improves Neutrophil-Dependent Airway Inflammation in Mice. Front Immunol (2020) 11:566953. doi: 10.3389/fimmu.2020.566953 33123138 PMC7566412

[28] Centurione L Di Baldassarre A Zingariello M Bosco D Gatta V Rana RA. Increased and Pathologic Emperipolesis of Neutrophils Within Megakaryocytes Associated With Marrow Fibrosis in GATA-1low Mice. Blood (2004) 104:3573–80. doi: 10.1182/blood-2004-01-0193 15292068

[29] Vyas P Ault K Jackson CW Orkin SH Shivdasani RA. Consequences of GATA-1 Deficiency in Megakaryocytes and Platelets. Blood (1999) 93:2867–75. doi: 10.1182/blood.V93.9.2867 10216081

[30] Vannucchi AM Bianchi L Cellai C Paoletti F Rana RA Lorenzini R. Development of Myelofibrosis in Mice Genetically Impaired for GATA-1 Expression (GATA-1low Mice). Blood (2002) 100(4):1123–32. doi: 10.1182/blood-2002-06-1913 12149188

[31] Zingariello M Martelli F Ciaffoni F Masiello F Ghinassi B D’Amore E. Characterization of the TGF-β1 Signaling Abnormalities in the Gata1low Mouse Model of Myelofibrosis. Blood (2013) 121(17):3345–63. doi: 10.1182/blood-2012-06-439661 PMC3637011 23462118

[32] Zingariello M Verachi P Gobbo F Martelli F Falchi M Mazzarini M. Resident Self-Tissue of Proinflammatory Cytokines Rather Than Their Systemic Levels Correlates With Development of Myelofibrosis in Gata1low Mice. Biomolecules (2022) 12(2):234. doi: 10.3390/biom12020234 35204735 PMC8961549

[33] Martelli F Ghinassi B Panetta B Alfani E Gatta V Pancrazzi. Variegation of the Phenotype Induced by the Gata1low Mutation in Mice of Different Genetic Backgrounds. Blood (2005) 106:4102–13. doi: 10.1182/blood-2005-03-1060 16109774

[34] Cavalieri B Mosca M Ramadori P Perrelli M-G De Simone L Colotta F. Neutrophil Recruitment in the Reperfused-Injured Rat Liver was Effectively Attenuated by Repertaxin, a Novel Allosteric Noncompetitive Inhibitor of CXCL8 Receptors: A Therapeutic Approach for the Treatment of Post-Ischemic Hepatic Syndromes. Int J Immunopathol Pharmacol (2005) 18(3):475–86. doi: 10.1177/039463200501800307 16164828

[35] Brandolini L Benedetti E Ruffini PA Russo R Cristiano L Antonosante A. CXCR1/2 Pathways in Paclitaxel-Induced Neuropathic Pain. Oncotarget (2017) 8(14):23188–201. doi: 10.18632/oncotarget.15533 PMC5410296 28423567

[36] Di Sapia R Zimmer TS Kebede V Balosso S Ravizza T Sorrentino D. CXCL1-CXCR1/2 Signaling is Induced in Human Temporal Lobe Epilepsy and Contributes to Seizures in a Murine Model of Acquired Epilepsy. Neurobiol Dis (2021) 158:105468. doi: 10.1016/j.nbd.2021.105468 34358616

[37] Migliaccio AR Rana RA Sanchez M Lorenzini R Centurione L Bianchi L. GATA-1 as a Regulator of Mast Cell Differentiation Revealed by the Phenotype of the GATA-1low Mouse Mutant. J Exp Med (2003) 197(3):281–96. doi: 10.1084/jem.20021149 PMC2193836 12566412

[38] Varricchio L Iancu-Rubin C Upadhyaya B Zingariello M Martelli F Verachi P. TGF-β1 Protein Trap AVID200 Beneficially Affects Hematopoiesis and Bone Marrow Fibrosis in Myelofibrosis. JCI Insight (2021) 6(18):e145651. doi: 10.1172/jci.insight.145651 34383713 PMC8492354

[39] Gilles L Arslan AD Marinaccio C Wen QJ Arya P McNulty M. Downregulation of GATA1 Drives Impaired Hematopoiesis in Primary Myelofibrosis. J Clin Invest (2017) 127(4):1316–20. doi: 10.1172/jci82905 PMC5373858 28240607

[40] Zhan H Ma Y Lin CHS Kaushansky K. JAK2V617F-Mutant Megakaryocytes Contribute to Hematopoietic Stem/progenitor Cell Expansion in a Model of Murine Myeloproliferation. Leukemia (2016) 30(12):2332–41. doi: 10.1038/leu.2016.114 PMC5158308 27133820

[41] Abbonante V Di Buduo CA Gruppi C Malara A Gianelli U Celesti G. Thrombopoietin/TGF-β1 Loop Regulates Megakaryocyte Extracellular Matrix Component Synthesis. Stem Cells (2016) 34(4):1123–33. doi: 10.1002/stem.2285 26748484

[42] Migliaccio AR Hoffman R. An Outline of the Outset of Thrombopoiesis in Human Embryos at Last. Cell Stem Cell (2021) 28(3):363–5. doi: 10.1016/j.stem.2021.02.007 PMC10450206 33667354

[43] Sun S Jin C Si J Lei Y Chen K Cui Y. Single-Cell Analysis of Ploidy and the Transcriptome Reveals Functional and Spatial Divergency in Murine Megakaryopoiesis. Blood (2021) 138(14):1211–24. doi: 10.1182/blood.2021010697 PMC8499048 34115843

[44] Wang H He J Xu C Chen X Yang H Shi S. Decoding Human Megakaryocyte Development. Cell Stem Cell (2021) 28(3):535–49.e8. doi: 10.1016/j.stem.2020.11.006 33340451

[45] Sim X Jarocha D Hayes V Hanby HA Marks MS Camire RM. Identifying and Enriching Platelet-Producing Human Stem Cell-Derived Megakaryocytes Using Factor V Uptake. Blood (2017) 130(2):192–204. doi: 10.1182/blood-2017-01-761049 28455282 PMC5510789

[46] Goldstein LJ Perez RP Yardley D Han LK Reuben JM Gao H. A Window-of-Opportunity Trial of the CXCR1/2 Inhibitor Reparixin in Operable HER-2-Negative Breast Cancer. Breast Cancer Res (2020) 22(1):4. doi: 10.1186/s13058-019-1243-8 31924241 PMC6954543

[47] Maffi P Lundgren T Tufveson G Rafael E Shaw JAM Liew A. Targeting CXCR1/2 Does Not Improve Insulin Secretion After Pancreatic Islet Transplantation: A Phase 3, Double-Blind, Randomized, Placebo-Controlled Trial in Type 1 Diabetes. Diabetes Care (2020) 43(4):710–8. doi: 10.2337/dc19-1480 PMC7876579 32019854

[48] Opfermann P Derhaschnig U Felli A Wenisch J Santer D Zuckermann A. A Pilot Study on Reparixin, a CXCR1/2 Antagonist, to Assess Safety and Efficacy in Attenuating Ischaemia-Reperfusion Injury and Inflammation After on-Pump Coronary Artery Bypass Graft Surgery. Clin Exp Immunol (2015) 180(1):131–42. doi: 10.1111/cei.12488 PMC4367101 25402332

[49] Cai X Li Z Zhang Q Qu Y Xu M Wan X. CXCL6-EGFR-Induced Kupffer Cells Secrete TGF-β1 Promoting Hepatic Stellate Cell Activation *via* the SMAD2/BRD4/C-MYC/EZH2 Pathway in Liver Fibrosis. J Cell Mol Med (2018) 22(10):5050–61. doi: 10.1111/jcmm.13787 PMC6156397 30106235

[50] Teijeira A Garasa S Ochoa MDC Cirella A Olivera I Glez-vaz J. Differential Interleukin-8 Thresholds for Chemotaxis and Netosis in Human Neutrophils. Eur J Immunol (2021) 51(9):2274–80. doi: 10.1002/eji.202049029 33963542

[51] Pariser DN Hilt ZT Ture SK Blick-Nitko SK Looney MR Cleary SJ. Lung Megakaryocytes are Immune Modulatory Cells. J Clin Invest (2021) 131(1):e137377. doi: 10.1172/JCI137377 PMC7773372 33079726

[52] Malara A Currao M Gruppi C Celesti G Viarengo G Buracchi C. Megakaryocytes Contribute to the Bone Marrow-Matrix Environment by Expressing Fibronectin, Type IV Collagen, and Laminin. Stem Cells (2014) 32(4):926–37. doi: 10.1002/stem.1626 PMC4096110 24357118

[53] Kuter DJ Gminski DM Rosenberg RD. Transforming Growth Factor Beta Inhibits Megakaryocyte Growth and Endomitosis. Blood (1992) 79(3):619–26. doi: 10.1182/blood.V79.3.619.bloodjournal793619 1732007

[54] Bresnick EH Lee HY Fujiwara T Johnson KD Keles S. GATA Switches as Developmental Drivers. J Biol Chem (2010) 285(41):31087–93. doi: 10.1074/jbc.R110.159079 PMC2951181 20670937

[55] Yue L Bartenstein M Zhao W Ho WT Han Y Murdun C. Efficacy of ALK5 Inhibition in Myelofibrosis. JCI Insight (2017) 2(7):e90932. doi: 10.1172/jci.insight.90932 28405618 PMC5374075

[56] McDevitt MA Shivdasani RA Fujiwara Y Yang H Orkin SH. A "Knockdown" Mutation Created by Cis-Element Gene Targeting Reveals the Dependence of Erythroid Cell Maturation on the Level of Transcription Factor GATA-1. Proc Natl Acad Sci (1997) 94(13):6781–5. doi: 10.1073/pnas.94.13.6781 PMC21235 9192642

[57] Migliaccio AR Martelli F Verrucci M Sanchez M Valeri M Migliaccio G. Gata1 Expression Driven by the Alternative HS2 Enhancer in the Spleen Rescues the Hematopoietic Failure Induced by the Hypomorphic Gata1low Mutation. Blood (2009) 114(10):2107–20. doi: 10.1182/blood-2009-03-211680 PMC2744572 19571316

[58] Wen QJ Yang Q Goldenson B Malinge S Lasho T Schneider RK. Targeting Megakaryocytic-Induced Fibrosis in Myeloproliferative Neoplasms by AURKA Inhibition. Nat Med (2015) 21(12):1473–80. doi: 10.1038/nm.3995 PMC4674320 26569382

