# Supplementary figures and images for: The CXCR1/CXCR2 Inhibitor Reparixin Alters the Development of Myelofibrosis in the Gata1 low Mice
Source: Front Oncol. 2022 Mar 22;12:853484. doi: 10.3389/fonc.2022.853484 (PMC8982152; doi:10.3389/fonc.2022.853484)

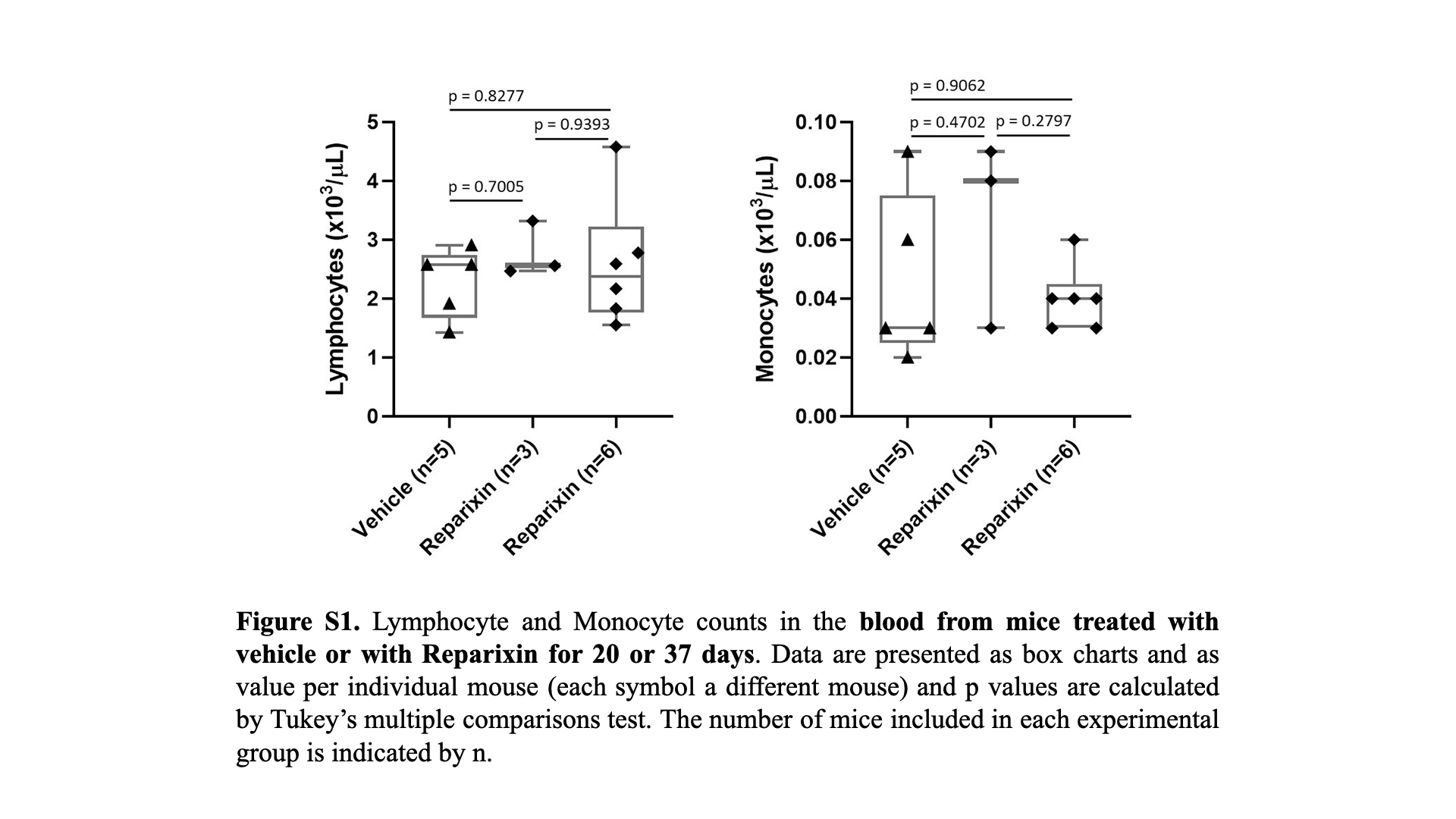

Supplement: Supplementary file 1 [file Image_1.tiff]

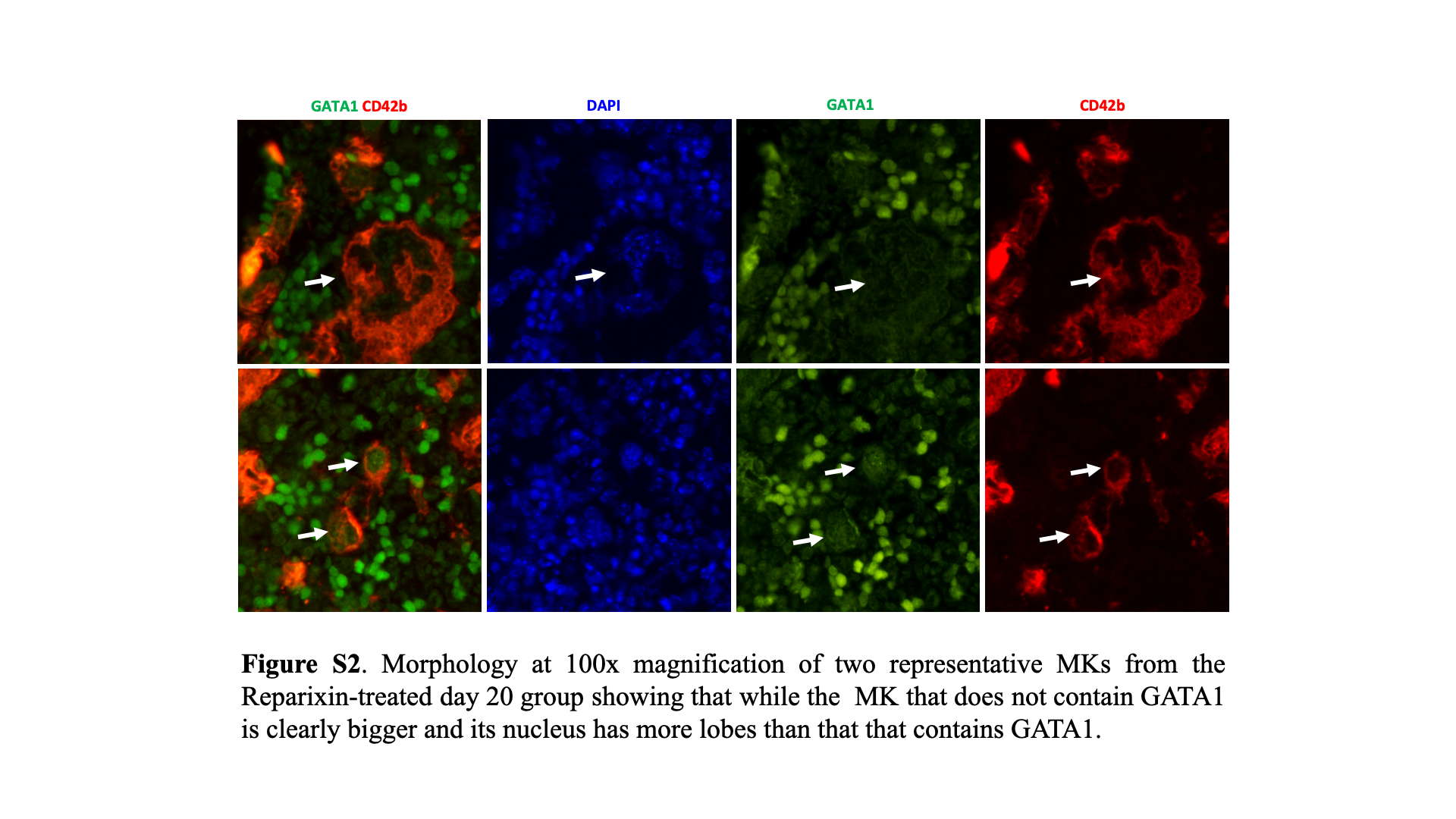

Supplement: Supplementary file 2 [file Image_2.tiff]
